# Supplementary material for: A multi-scale finite element method for investigating fiber remodeling in hypertrophic cardiomyopathy
Source: Sci Rep. 2025 Aug 30;15:31961. doi: 10.1038/s41598-025-17778-5 (PMC12398582; doi:10.1038/s41598-025-17778-5)
Supplement: Supplementary file 1 — Supplementary Material 1 [file 41598_2025_17778_MOESM1_ESM.docx]

**Supplementary Materials**

**Supplementary tables**

Table S1. Model parameters for baseline simulation.

| Component | Parameter | Value | Units |
| --- | --- | --- | --- |
| Lumped parameter model of systemic circulation | HR | 65 (bpm) | (bpm) |
|  | V_total_ | 4.5 (liters) | (liters) |
|  | R_aorta_ | 25.0 | (mmHg L^-1^ s) |
|  | R_arteries_ | 25.0 | (mmHg L^-1^ s) |
|  | R_arterioles_ | 787.0 | (mmHg L^-1^ s) |
|  | R_capillaries_ | 350.0 | (mmHg L^-1^ s) |
|  | R_venules_ | 50.0 | (mmHg L^-1^ s) |
|  | R_veins_ | 50.0 | (mmHg L^-1^ s) |
|  | R_LV_ | 10.0 | (mmHg L^-1^ s) |
|  | C_aorta_ | 0.00035 | (mmHg^-1^ L s^-1^) |
|  | C_arteries_ | 0.0008 | (mmHg^-1^ L s^-1^) |
|  | C_arterioles_ | 0.001 | (mmHg^-1^ L s^-1^) |
|  | C_capillaries_ | 0.01 | (mmHg^-1^ L s^-1^) |
|  | C_venules_ | 0.03 | (mmHg^-1^ L s^-1^) |
|  | C_veins_ | 0.07 | (mmHg^-1^ L s^-1^) |
|  | V_aorta,slack_ | 0.3 | (liters) |
|  | V_arteries,slack_ | 0.3 | (liters) |
|  | V_arterioles,slack_ | 0.1 | (liters) |
|  | V_capillaries,slack_ | 0.25 | (liters) |
|  | V_venules,slack_ | 0.5 | (liters) |
|  | V_veins,slack_ | 2.0 | (liters) |
| Myocardium passive properties | C | 200.0 | (Pa) |
|  | b_ff_ | 8.0 | (Unitless) |
|  | b_xx_ | 3.58 | (Unitless) |
|  | b_fs_ | 1.63 | (Unitless) |
|  | C_1_ | 250.0 | (Pa) |
|  | C_2_ | 15.0 | (Unitless) |
| MyoSim model of contraction | k_on_ | 1.7$\times$10^8^ | (M^-1^ s^-1^) |
|  | k_off_ | 200 | (s^-1^) |
|  | k_coop_ | 5 | (Unitless) |
|  | k_1_ | 3.7 | (s^-1^) |
|  | k_2_ | 200 | (s^-1^) |
|  | k_3_ | 100 | (s^-1^ nm^-1^) |
|  | k_4,0_ | 80 | (s^-1^) |
|  | k_4,1_ | 1.5 | (s^-1^ nm^-4^) |
|  | k_cb_ | 0.001 | (pN nm^-1^) |
|  | x_ps_ | 5 | (nm) |
|  | N_0_ | 6.9$\times$10^16^ | (m^-2^) |
|  | k_B_ | 1.38$\times$10^-23^ | (JK^-1^) |
|  | T | 310 | (K) |
| Two compartment model of Ca^2+^ transient | k_SERCA_ | 7.866 | (M^-1^ s^-1^) |
|  | k_act_ | 8.06$\times$10^-2^ | (M^-1^ s^-1^) |
|  | k_leak_ | 6$\times$10^-4^ | (M^-1^ s^-1^) |
| Fiber reorientation law | $\kappa$ | 4000 | (ms) |

**Supplementary figures:**


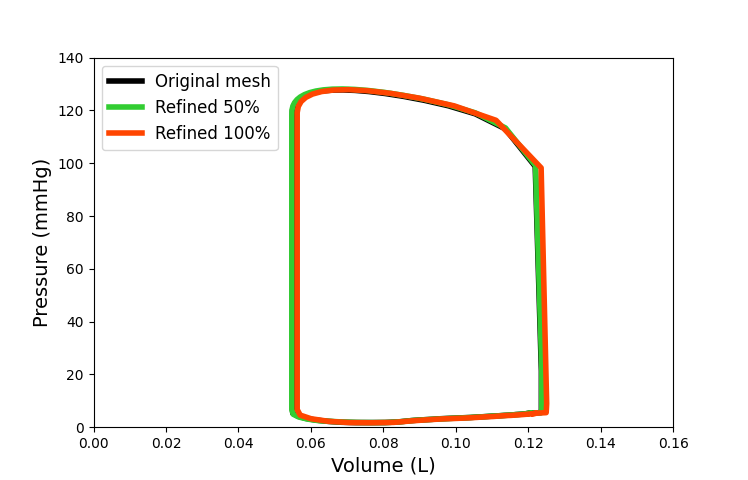


**Figure S1:** Comparison of Pressure-Volume (PV) loops in LV Models with different levels of mesh refinement. The original mesh contains ~1250 quadratic tetrahedral elements. Note that there is minimal variation between the loop plots.


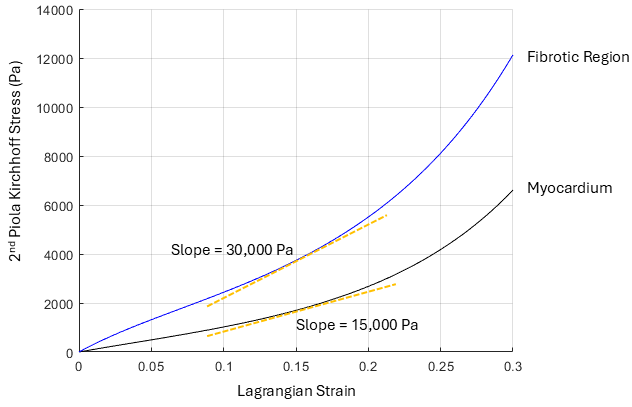


**Figure S2:** Stress-strain curves of the myocardium and fibrotic regions. These represent the mechanical response along the fiber direction. Stiffness was calculated by taking the slope of the stress-strain curves at a strain value of 0.15 (i.e., 15% strain).


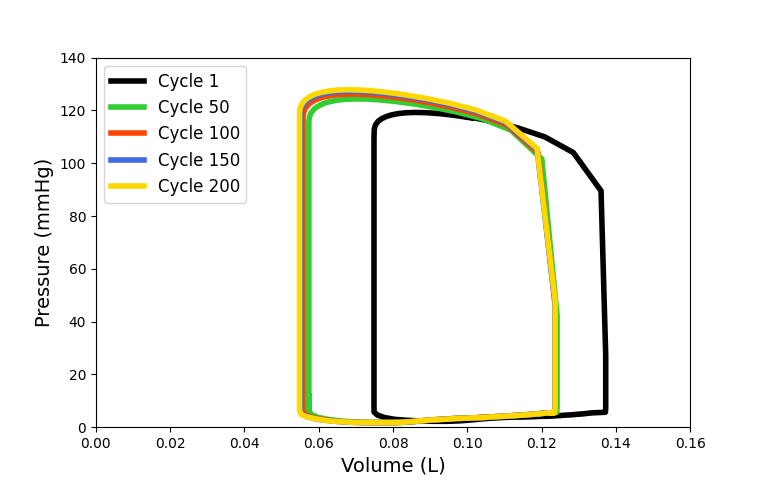


**Figure S3:** Evolution and convergence of Pressure-Volume (PV) loops in LV model. PV loops are plotted for the 1st, 50th, 100th, 150th and 200th cardiac cycles with fiber reorientation.


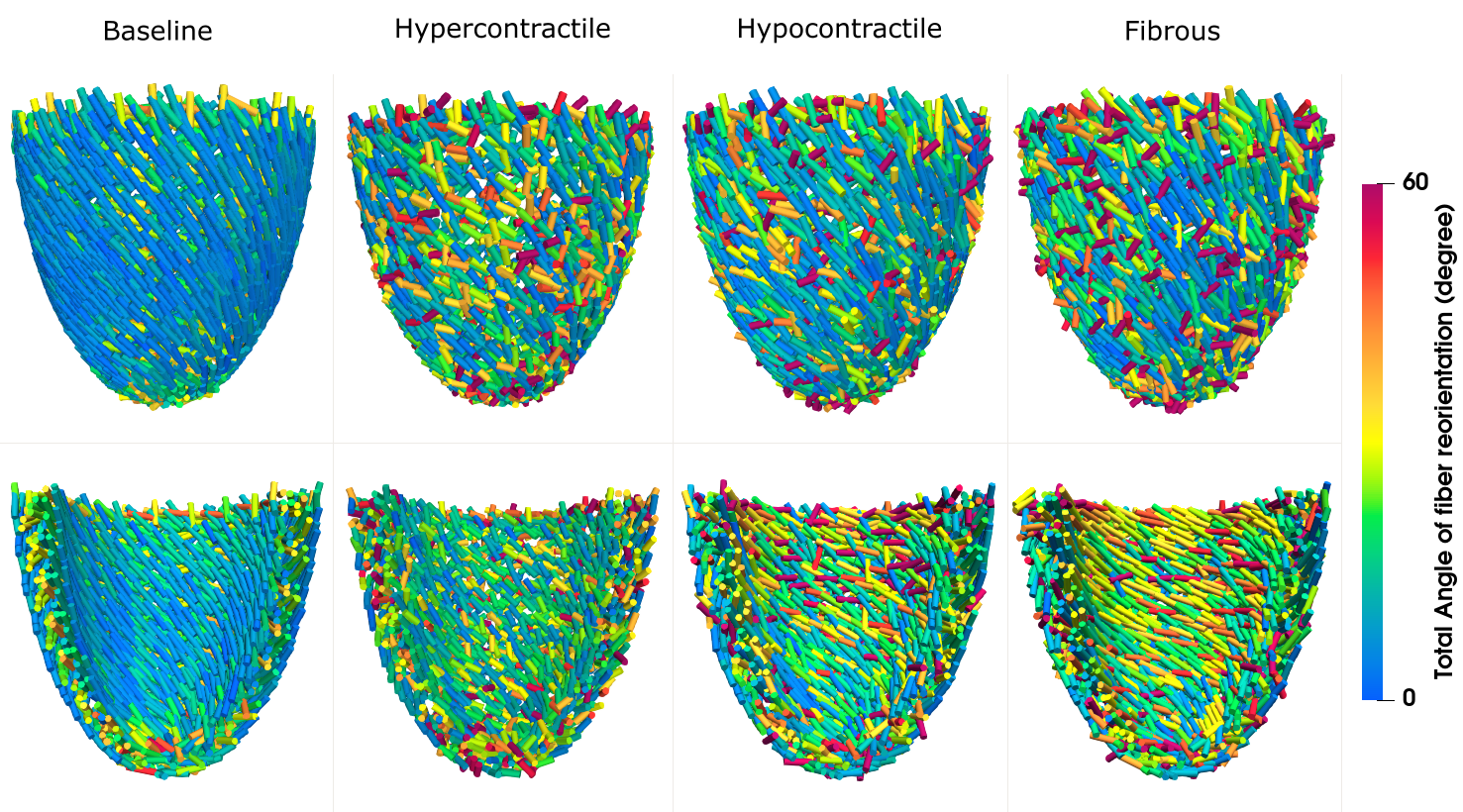


**Figure S4:** Comparison of fiber disarray between baseline and perturbed LV models based on the total angle of fiber reorientation. Top: Epicardial side of the myocardium. Bottom: Endocardial side of the myocardium.


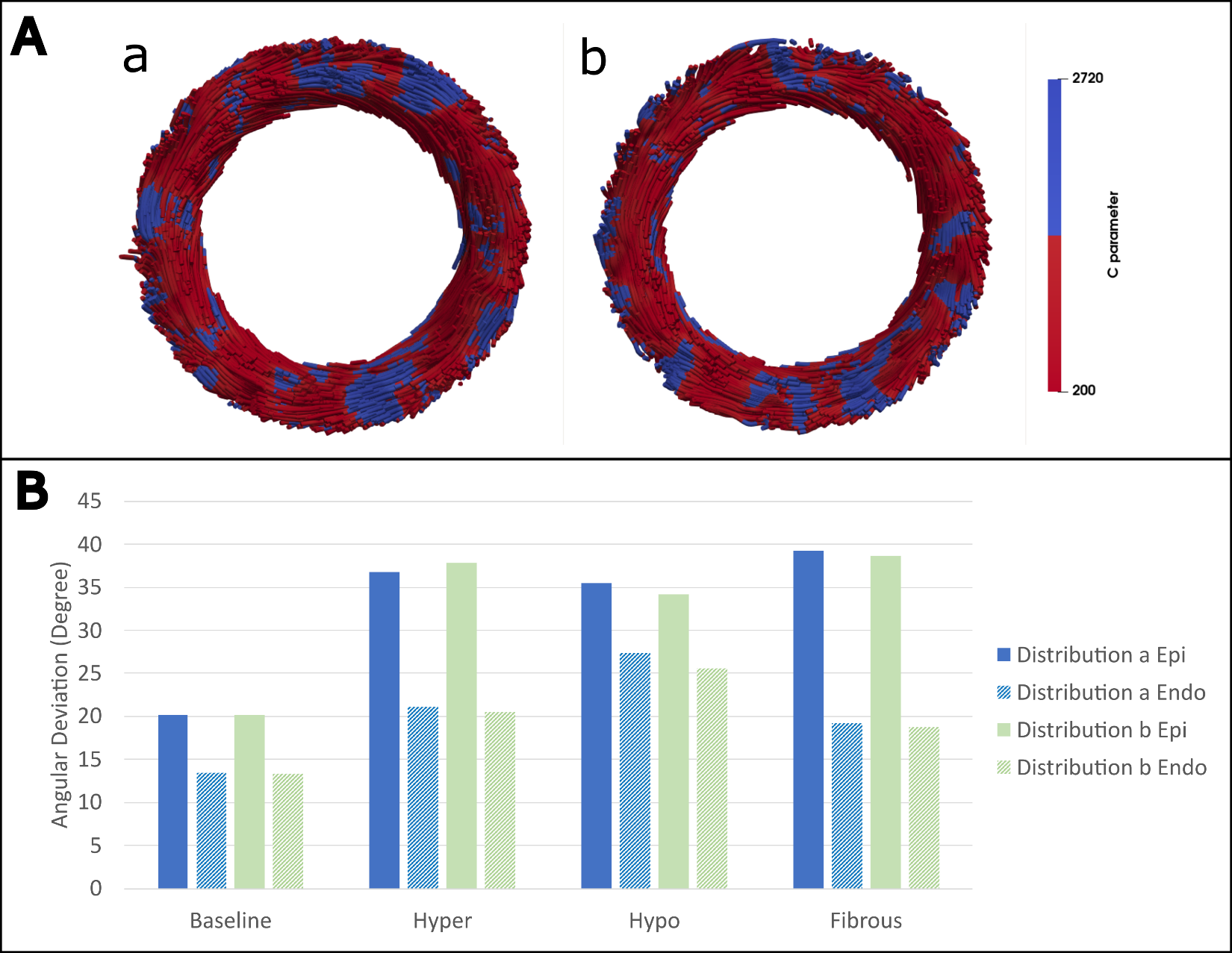


**Figure S5:** Spatial variability without variation in size of perturbed regions, i.e., the heterogenous perturbed tissue consisted of 30% of the LV myocardium and the minimum size of a perturbed region was 3 mm. **A:** Short axis view of mid ventricle with perturbed distribution of a and b. **B:** Induced disarray from distribution a and b in baseline and perturbed LV models.
